# The madness of HIV prevention trials

**DOI:** 10.1186/1745-6215-16-S2-I1

**Published:** 2015-11-16

**Authors:** S McCormack

**Affiliations:** 1 MRC Clinical Trials Unit at University College London and Imperial College London. London, UK

## 

In the opening plenary lecture, Prof McCormack discusses the trials and tribulations of HIV prevention.

